# Impact of Tail Loss on the Behaviour and Locomotor Performance of Two Sympatric *Lampropholis* Skink Species

**DOI:** 10.1371/journal.pone.0034732

**Published:** 2012-04-16

**Authors:** Gillian L. Cromie, David G. Chapple

**Affiliations:** School of Biological Sciences, Monash University, Clayton, Victoria, Australia; Macquarie University, Australia

## Abstract

Caudal autotomy is an anti-predator behaviour that is used by many lizard species. Although there is an immediate survival benefit, the subsequent absence of the tail may inhibit locomotor performance, alter activity and habitat use, and increase the individuals' susceptibility to future predation attempts. We used laboratory experiments to examine the impact of tail autotomy on locomotor performance, activity and basking site selection in two lizard species, the delicate skink (*Lampropholis delicata*) and garden skink (*L. guichenoti*), that occur sympatrically throughout southeastern Australia and are exposed to an identical suite of potential predators. Post-autotomy tail movement did not differ between the two *Lampropholis* species, although a positive relationship between the shed tail length and distance moved, but not the duration of movement, was observed. Tail autotomy resulted in a substantial decrease in sprint speed in both species (28–39%), although this impact was limited to the optimal performance temperature (30°C). Although *L. delicata* was more active than *L. guichenoti*, tail autotomy resulted in decreased activity in both species. Sheltered basking sites were preferred over open sites by both *Lampropholis* species, although this preference was stronger in *L. delicata*. Caudal autotomy did not alter the basking site preferences of either species. Thus, both *Lampropholis* species had similar behavioural responses to autotomy. Our study also indicates that the impact of tail loss on locomotor performance may be temperature-dependent and highlights that future studies should be conducted over a broad thermal range.

## Introduction

Autotomy, the capacity to ‘voluntarily’ shed a limb or appendage, is an antipredator behaviour that has evolved independently in numerous vertebrate (e.g. reptiles, amphibians, fishes) and invertebrate groups (e.g. insects, spiders, crustaceans, echinoderms) (reviewed in [1], [2]). This defensive tactic has been most widely studied in lizards where caudal autotomy occurs in at least 13 of the 20 recognised families [2], [3]. Tail autotomy is generally employed as a last ditch strategy in lizards, after other antipredator behaviours such as crypsis and fleeing have failed [2], [4], [5]. The detachment of the tail enables the lizard to escape from the predators' grasp, and the post-autotomy thrashing of the tail acts to distract the predator while it makes its getaway [6]–[8].

Many lizard species have fracture planes within the majority of their caudal vertebrae, enabling individuals to shed their tail at any point along its length [2], [9]. Whilst longer shed tail portions move further and have been shown to be more effective at distracting predators [10], it takes the individual longer to replace the tail [2]. In addition, lizard species often store fat in their tail and the amount of energy reserves lost during autotomy may be directly related to the length of tail discarded [11]–[13]. In general, the costs associated with caudal autotomy are transient and persist only until a substantial proportion of the tail has been regenerated [3], [14].

Following tail loss, lizards are unable to employ caudal autotomy as a defensive tactic again until they have sufficiently regrown their tail, and are therefore more reliant on antipredator behaviours such as fleeing [2], [4]. However, in the vast majority of species, tail autotomy acts to decrease stride length [15] and results in a substantial reduction in locomotor performance (e.g. [3], [10], [16]–[19]). This reduced mobility and enhanced predation risk may influence the behaviour of lizards during tail regeneration. Lizards may be less active following autotomy, increase their use of sheltered microhabitats, and remain closer to cover or refuge [3], [16], [19]–[23]. These behaviours may decrease an individual's exposure and vulnerability to predation, but might also influence their thermoregulatory behaviour, foraging ability, and speed of tail regeneration [20], [24], [25].

**Figure 1:**
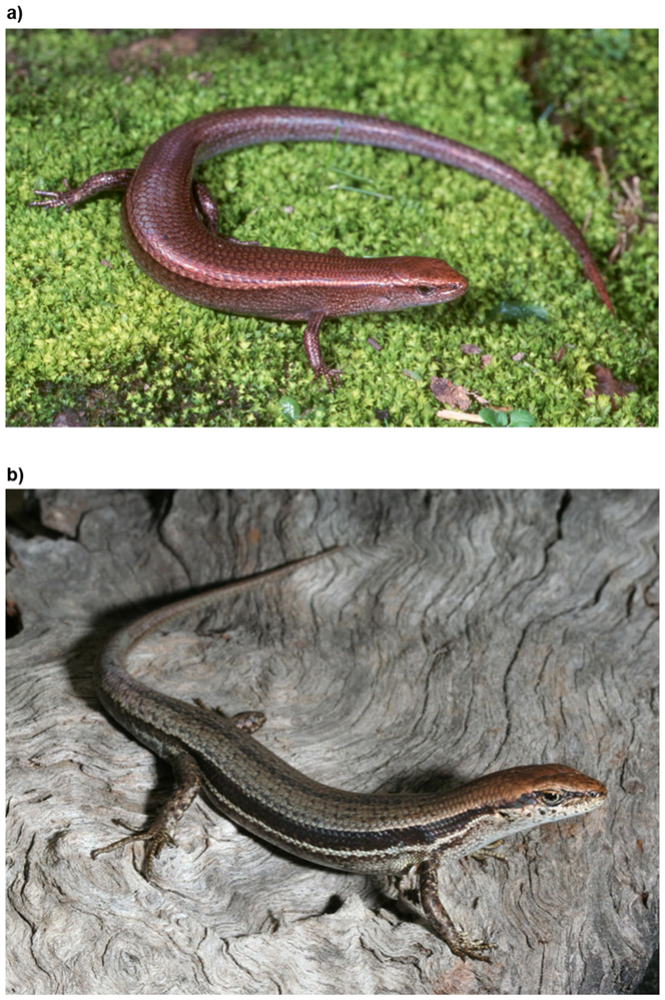
The two study species: a) delicate skink (*Lampropholis delicata*), and b) garden skink (*Lampropholis guichenoti*). Photographs: Nick Clemann.

Here we use laboratory experiments to investigate the locomotor performance, activity and basking site preferences of two sympatric species, the delicate skink (*Lampropholis delicata* De Vis) and the garden skink (*L. guichenoti* Duméril and Bibron) (Figure 1). Specifically, we aimed to compare: i) the baseline behaviour of the two species, and ii) the behavioural response of each species to tail loss. The two *Lampropholis* species are near identical in body size (∼35–55 mm adult snout-vent length [SVL]) and life history (e.g. oviparous, clutch size, reproductive ecology), and are both diurnal heliotherms that inhabit leaf litter and ground debris [26], [27]. *Lampropholis delicata* has a broader distribution, occurring in rainforest, wet/dry sclerophyll forests, woodlands and heaths in eastern Australia from north Queensland to southern Tasmania [27]. *Lampropholis guichenoti* occurs in sympatry with *L. delicata* throughout the vast majority of its range (southeast Queensland to southern Victoria), but prefers drier and more open microhabitats [27]–[29]. Both species thrive in suburban habitats throughout southeastern Australia [27], [30].

Given that the two species are exposed to an identical suite of predators where they occur in sympatry, we predicted that they would exhibit similar behavioural responses to tail autotomy. However, since *L. delicata* is the only Australian lizard species that has successfully established and subsequently become invasive overseas (Hawaiian Islands, New Zealand, Lord Howe Island; [29], [31], [32]), we also sought to investigate whether it exhibited a divergent response to caudal autotomy that might contribute to its success as an invasive species.

## Materials and Methods

### Collection and housing

Since gravidity (females of both species were gravid during the study period; [26]) is known to influence behaviour in *Lampropholis* skinks [33], we only used adult males (i.e. SVL>35 mm) with full length tails (tail length>SVL) for our experiments. We collected the two *Lampropholis* species (28 *L. delicata*, 29 *L. guichenoti*) by hand from Newtown in suburban Sydney (33°53′39″S, 151°10′44″E) in October 2009. The collection area represents one of the known source regions for successful *L. delicata* introductions (DGC, unpublished data). Both species were abundant at the site, and were frequently observed to bask together [29].

The lizards were transported to Monash University and housed in clear plastic containers (42 L×31 W×23 cm H) in a constant temperature room (20±1°C) with a 14 L∶ 10 D photoperiod (0600–2000 h). The housing containers were lined with newspaper and each included a plastic shelter site and two terracotta basking tiles positioned under a heat lamp that was activated for 10 h each day (0800–1800 h). This created a thermal gradient (20–38°C) within each container and enabled the skinks to thermoregulate freely. The lizards were fed three times weekly with crickets (*Acheta domesticus*) dusted with reptile supplement (Reptivite™) and provided with water *ad libitum*. Since *Lampropholis* skinks modify their behaviour following large meals [33], the lizards were not fed in the 24 h prior to each behavioural trial.

### Experimental procedures and timeline

Within each species, lizards were randomly assigned to either a control (14 *L. delicata*, 14 *L. guichenoti*) or experimental group (14 *L. delicata*, 15 *L. guichenoti*). A two-way ANOVA confirmed that body size (SVL) and tail length did not differ between individuals assigned to each group, or between the two species (Table 1). All analyses in the study were performed in R v2.10.1 [34].

**Table 1:** Body size (snout-vent length, SVL) and tail length of the *Lampropholis delicata* and *L. guichenoti* assigned to the control and experimental groups.

Species	SVL (mm)				Tail Length (mm)			
	Mean ± SE	ANOVA			Mean ± SE	ANOVA		
		Factor	F	*P*		Factor	F	*P*
***L. delicata***								
Control	40.57±0.45	**Treatment:**	F_1,53_ = 1.13	0.29	55.29±2.84	**Treatment:**	F_1,53_ = 0.31	0.58
Experimental	40.29±0.34	**Species:**	F_1,53_ = 0.29	0.59	53.64±2.50	**Species:**	F_1,53_ = 1.57	0.22
***L. guichenoti***		**Interaction:**	F_1,53_ = 2.35	0.13		**Interaction:**	F_1,53_ = 0.67	0.42
Control	40.53±0.48				57.47±1.56			
Experimental	40.79±0.36				57.07±2.08			

Behavioural experiments were conducted over an eight-week period. Upon arrival in the laboratory, baseline measurements were taken of sprinting performance, activity in an open environment, and basking site preference. Following the inducement of tail autotomy in the experimental groups, the sprinting performance (2–7 days post-autotomy), activity (9–13 days post-autotomy) and basking site preferences (12–17 days post-autotomy) of all lizards (both control and experimental groups) were re-assessed to determine the impact of tail loss on the behaviour of both *Lampropholis* species.

**Table 2:** Duration of post-autotomy tail movement (± SE) in *Lampropholis delicata* and *L. guichenoti*, and the distance (± SE) that the shed tails moved.

	Species	
	*L. delicata*	*L. guichenoti*
Duration (sec)	171.78±10.91	191.95±12.00
Distance moved (transitions)	135.00±22.74	147.92±35.01

Intravertebral caudal autotomy was induced in the lizards assigned to the experimental group using blunt forceps in a room with an ambient temperature of 20°C. Caudal autotomy in lizards is under neurological control and can only be performed by conscious animals [2], [4]. Immediately following autotomy (two-thirds of the original tail length), the shed tail was placed into a large arena (40 L×30 W×30 cm H) marked with grid squares (2 cm×2 cm) to examine post-autotomy tail movement. When the shed tail stopped moving it was prodded with forceps (to imitate a predators touch or bite). If the tail did not recommence moving within 2 sec it was prodded again. The trial was ceased when the tail did not recommence movement after three consecutive prods. All trials were recorded using a digital video recorder and subsequently analysed to determine the time that the tail spent moving and the distance that it covered (taken as the number of grid squares it moved through, using the tail base as the point of reference). The length of each shed tail (mm) was recorded at the conclusion of each trial. All control animals were handled in the same manner as the experimental animals, except that tail autotomy was not induced.

Post-autotomy tail movement of the two species was compared using Analysis of Covariance (ANCOVA), with tail length as the covariate. Simple linear regression was conducted to investigate the relationship between the length of shed tail and both the distance moved and the time spent moving. All data were checked for normality and homogeneity of variances.

### Thermal sensitivity of locomotor performance

Although the sprinting performance of lizards is sensitive to the thermal environment and body temperature (reviewed in [35]), apart from one exception (e.g. [3]), studies have only examined the impact of tail loss within a limited temperature range. We investigated the sprinting performance of the two *Lampropholis* species across five different temperatures (15, 20, 25, 30 and 35°C). The thermal sensitivity of sprint speed was assessed in all lizards during both testing periods: initial baseline trials, and following the experimental procedures. Each lizard was run twice at each test temperature (30 min between each run), with at least 24 h between trials at different temperatures. To avoid any potential order effects, the order in which each lizard completed the five test temperatures was randomised.

Lizards were warmed or cooled to the test temperature in a custom built temperature-controlled metal chamber for 30 min prior to each trial. The lizards were then sprinted (using light taps on the tail/tail stump with a paintbrush) along a 1 m long racetrack (width 10 cm), set at the test temperature, with the speed of the lizards recorded by photodiode sensors located at 25 cm intervals along the length of the track. The times were logged onto a computer using customised software (WinTec Version: 5.3.3, Tain Electronics, Melbourne, Australia). Following their first run, the skinks were returned to the chamber and warmed/cooled for a further 30 min before being re-run at the same test temperature. Thus, for each individual we obtained their sprint speed over eight 25 cm intervals at each test temperature. The fastest interval time was considered to be the lizards maximal sprint speed at the particular temperature (e.g. [36]).

All data were checked for normality and homogeneity of variance, with sprint speed log transformed to normalize the data. To compare the baseline sprint performance of the two *Lampropholis* species, a mixed effects model was fitted with species and temperature as fixed effects and individual lizard ID as a random effect. To determine the impact of tail loss on sprint speed in both species, a linear mixed effects model with fixed effects for species, temperature and experimental group (control or tail loss) and a random effect for individual lizard ID. The analysis incorporated repeated measures since the same lizards were run at each of the five temperatures during both testing periods.

### Activity

The activity of the two *Lampropholis* species was examined in an open opaque-walled test arena (55 L×32 W×24 cm H), with 20 grid squares (8×11 cm) marked on the floor. All animals were tested during both testing periods, and trials were conducted at 20°C. The lizard was placed under a clear plastic holding container for 10 min prior to the commencement of the trail to acclimate to the test arena. At the start of the trial, the plastic container was removed and the lizard was able to freely move around the test arena for 45 min. Each trial was video recorded and during playback the activity of each lizard (taken as the number of times the lizard moved between grid squares) was recorded with the aid of Etholog v2.2.5 [37].

The activity of each species was compared using an independent t-test. The impact of tail loss on activity was investigated using a mixed effects model with species and treatment as fixed effects and individual ID as a random effect. Individual lizard ID was used to reduce variability in the model.

### Basking site selection

We examined the basking site selection of the two *Lampropholis* species to determine whether they preferred sheltered or open sites, and if this preference was modified following caudal autotomy. The trials were performed in large, opaque-walled test arenas (55 L×32 W×24 cm H). The test arena was divided into three equal sections (open basking site, no preference zone, sheltered basking site). The basking sites (either open or sheltered), positioned under a 40-W heating lamp, were placed at each end of the arena. The open basking site consisted of a flat basking tile (10×10 cm), while the sheltered basking site also had an opaque plastic shelter area (11 L×8 W×3 cm H) either side of the tile so that lizards could bask while under cover.

The lizard was placed in the neutral region under an open topped, clear plastic container 10 min prior to the commencement of the trial to enable it to acclimatise to the test arena. At the start of the trial, the plastic container was removed and the lizard was able to freely move and select one of the two basking sites. The temperature underneath the heat lamps (∼35°C) was substantially higher than the ambient temperature (20°C), prompting the lizards to use the basking sites. Each trial lasted for 45 min and was video recorded and subsequently analysed (with the aid of Etholog) to determine the time spent at each basking site.

The time spent in the sheltered basking site was arcsine square root transformed in all analyses. The time spent in the sheltered site was compared between the two species using an independent t-test. The impact of tail loss on basking site selection was examined using a mixed effects model with individual lizard ID as a random effect. Individual lizard ID was used to reduce variability in the model.

**Figure 2:**
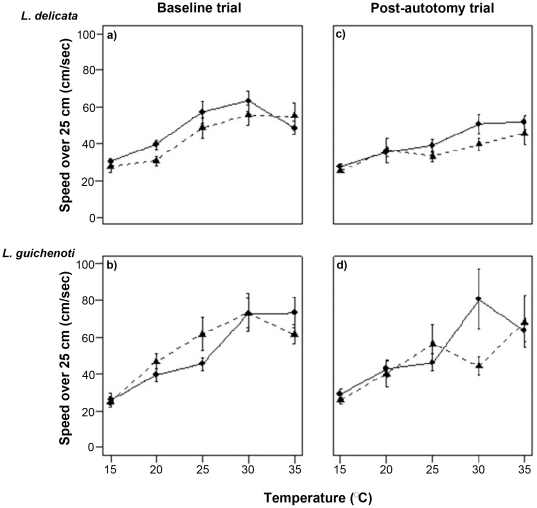
Impact of tail loss on maximal sprint speed in *Lampropholis delicata* and *L. guichenoti*. The control group is represented by a solid line and circles, while the experimental group is represented by a dashed line and triangles. The baseline trials are presented on the left (a, b), with the post-autotomy trials on the right (c, d). Error bars indicate ±1 SE.

## Results

### Post-autotomy tail movement

The post-autotomy tail movement of the two *Lampropholis* species did not differ, either in terms of the time spent moving (F_1,21_ = 0.548, *P* = 0.467) or the distance moved (F_1,21_ = 0.303, *P* = 0.588) (Table 2). However, there was a strong positive relationship between the length of the shed tail and the time spent moving (r^2^ = 0.237, t_22_ = 2.615, *P* = 0.016).

**Figure 3:**
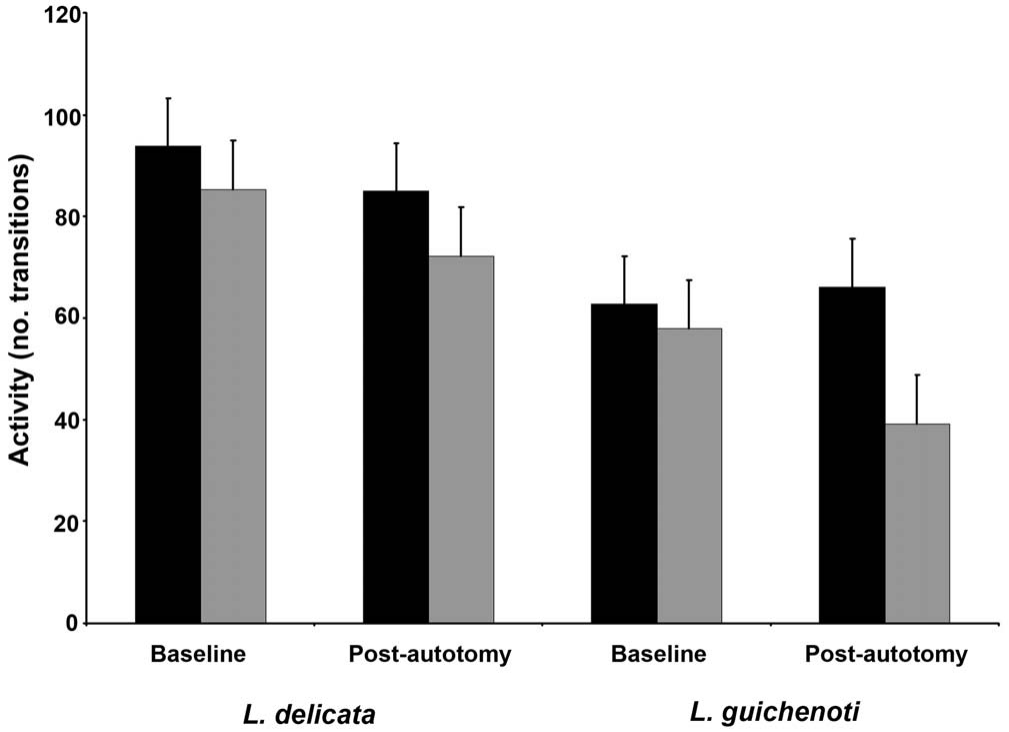
Activity (number of transitions between grid squares) of *Lampropholis delicata* and *L. guichenoti* prior to the experimental treatment (baseline trial), and following the inducement of caudal autotomy in the experimental group (post-autotomy trial). The control (black bars) and experimental groups (grey bars) within each species are indicated. Error bars indicate ±1 SE.

### Thermal sensitivity of locomotor performance

In the initial baseline trials, the sprinting performance of both *Lampropholis* species was strongly influenced by body temperature (F_1,4_ = 108.19, P<0.001), with the optimal sprint speed occurring at 30°C (Figure 2a,b). The baseline sprint speed of the two species was equivalent across most test temperatures (F_1,54_ = 0.766, *P* = 0.385), except that *L. guichenoti* ran faster at 35°C (F_1,54_ = 5.276, *P* = 0.026) (Figure 2a,b).

**Figure 4:**
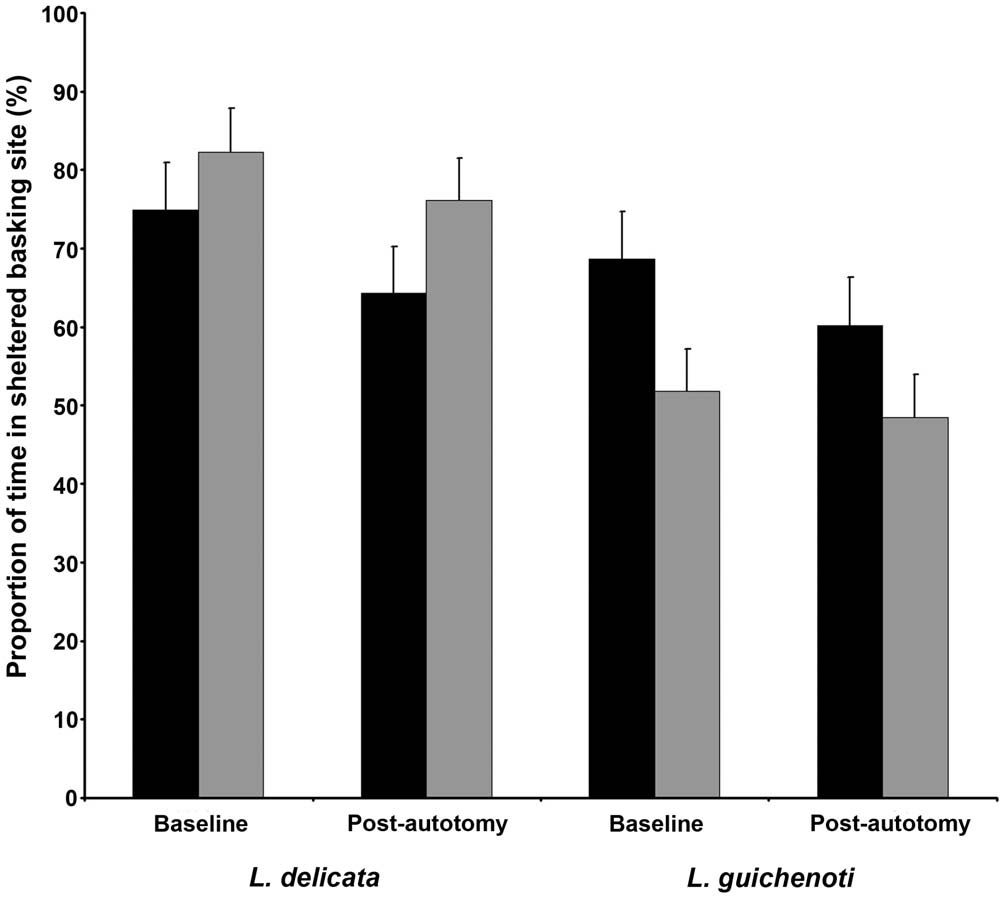
Proportion of time spent in the sheltered basking site by *Lampropholis delicata* and *L. guichenoti* prior to the experimental treatment (baseline trial), and following the inducement of caudal autotomy in the experimental group (post-autotomy trial). The control (black bars) and experimental groups (grey bars) within each species are indicated. Error bars indicate ±1 SE.

The influence of tail autotomy on locomotor performance was similar in both species (Interaction: F_4,208_ = 1.290, *P* = 0.275) (Figure 2a–d). Although tail loss reduced sprint speed in both *L. delicata* and *L. guichenoti*, the magnitude of this trend was dependent on the test temperature and a statistically significant reduction was only observed at the optimal performance temperature (30°C; F_1,52_ = 9.79, *P* = 0.003) (Figure 2a–d).

### Activity

In the baseline trials, *L. delicata* was more active than *L. guichenoti* (t = 2.92, df = 55, *P* = 0.005) (Figure 3). Tail loss acted to reduce activity relative to the control animals (F_1,54_ = 4.14, *P* = 0.04), a response which was consistent across both species (F_1,54_ = 0.51, *P* = 0.47) (Figure 3). However, following caudal autotomy, *L. delicata* remained more active than *L. guichenoti* (F_1,54_ = 6.74, *P* = 0.01) (Figure 3).

### Basking site preference

Although both *Lampropholis* species exhibited a preference for the sheltered basking sites in the baseline trials, this preference was stronger in *L. delicata* (t = 2.61, df = 54, *P* = 0.01) (Figure 4). Following autotomy, neither *L. delicata* (F_1,27_ = 3.18, *P* = 0.09) or *L. guichenoti* (F_1,27_ = 3.21, *P* = 0.08) differed in their basking site preferences compared to their tailed conspecifics, although the stronger tendency for *L. delicata* to bask in sheltered sites (relative to *L. guichenoti*) was maintained following autotomy (t = 2.50, df = 25, *P* = 0.004) (Figure 4).

## Discussion

Our study indicates that the two sympatric *Lampropholis* lizard species exhibit a similar behavioural response to tail autotomy. This result is consistent with our predictions, and may be due to the similar habitat preferences [27] and suite of potential predators of the two congeners. However, we did not examine whether the two species differed in the amount of tail shed during predatory encounters [11], [38], or the ease with which this autotomy occurred [2], [4]. Our post-autotomy tail movement trials, which standardised the position of autotomy (two-thirds tail loss), indicated that there was a positive relationship in both species between the length of tail shed and the distance moved, but not the time spent moving. A similar result was recently reported in the speckle-lipped mabuya (*Trachylepis maculilabris*) where longer tail portions (i.e. two-thirds tail, complete tail loss) moved further, but not for a longer duration, than shorter tail segments (i.e. one-third tail) [10]. This might be a consequence of the rate at which lactate accumulates in the autotomised tail being independent of its length [7], [10]. Thus, lizards may be constrained in their capacity to increase the duration of post-autotomy tail movement, but are still able to shed longer tail segments to enhance the effectiveness of predator distraction [6], [8].

Tail autotomy resulted in a substantial decrease in sprinting performance in both *L. delicata* (28% reduction) and *L. guichenoti* (39% reduction). However, the impaired locomotor ability was only evident at the optimal performance temperature for both *Lampropholis* species (30°C). The reduction in sprint speed immediately following autotomy was similar to that reported previously in other lizard species: *Cnemidophorus sexlineatus* (36% at 35–41°C [39]), *Cophosaurus texanus* (32% at 34–41°C; [40]), *Uma notata* (42% at 34–41°C; [40]), *Scincella lateralis* (35%; [16]), *Psammodromus algirus* (40%; [15]), *Niveoscincus metallicus* (33% at 26°C; [17]), *Takydromus septentrionalis* (43% at 26°C; [14]), *Eumeces fasciatus* (23%; [41]), *Sceloporus virgatus* (43%; [18]), *Trachylepis maculilabris* (41–42% at 28°C; [10]). However, these studies only examined sprinting performance at the optimal temperature, or within the preferred temperature range. We are only aware of one other study [3] that examined the impact of tail autotomy on locomotor performance across a broad range of temperatures. This study reported a relatively minor decrease (12–15%) in the sprinting performance of *Lampropholis guichenoti* at warmer temperatures (20.5–32.5°C), but no significant reduction at cooler temperatures (16°C). In *L. guichenoti*, the impaired locomotor performance only persisted for 40 days post-autotomy, when the lizards had regrown almost half of their original tail length [3].

The tail has an important role in enhancing the sigmoidal movement that many skinks exhibit at their maximal sprint speed [17]. When the tail is absent skinks are unable to generate the same degree of lateral movement and rely more on their limbs for propulsion [15], [17]. In essence, tailless lizards employ a gait that is predominately used by individuals at slower speeds and thus need to work harder (i.e. increase stride frequency) to maintain equivalent speeds to tailed lizards [15], [17], [42]. This increased exertion has been demonstrated to decrease stamina in some species (e.g. *Niveoscincus metallicus*; [17]) and might not be possible in all situations. Since sigmoidal movement is most regularly employed by *Lampropholis* skinks at optimal performance temperatures (our observations), the reduction in sprint speed is likely to be most pronounced at these temperatures rather than suboptimal temperatures. Thus, future studies investigating the impact of caudal autotomy on locomotor performance should examine sprint speed across a broad range of test temperatures.

*Lampropholis delicata* is a successful invasive species that is known to be more exploratory than *L. guichenoti* [29]. The higher activity of *L. delicata* in our baseline trials is consistent with this previous research. However, as predicted, our study demonstrates that both *Lampropholis* species reduce their activity following tail autotomy. Similar reductions in activity have been reported previously in lizards. For example, tailless males of the Iberian rock lizard (*Lacerta monticola*) are less active, move shorter distances, and pause more frequently than individuals with complete tails [21]. Similarly, ground skinks (*Scincella lateralis*) decrease their activity under laboratory conditions following autotomy [16]. Such reduction in activity following tail loss is presumed to be due to a combination of reduced locomotor performance and increase vulnerability to predation [2].

A previous study on *Lampropholis guichenoti* demonstrated that individuals sheltering in more open habitats (grass versus rocks & logs) have an enhanced risk of predation [3]. In our study, both *Lampropholis* species displayed a preference for basking in sheltered locations, although this tendency was significantly stronger in *L. delicata*. This result is consistent with the known microhabitat preferences of each species [27], [28]. Although tail loss had no impact on basking site preferences, the initial difference between the two species was maintained following autotomy, with tailless *L. guichenoti* more likely to bask in exposed areas compared to tailless *L. delicata*. Although tailless *L. guichenoti* tend to flee earlier than tailed individuals in response to an approaching predator [3], this behaviour may not compensate for their increased vulnerability in open habitats. In contrast, the tendency for the invasive *L. delicata* to bask in sheltered areas may decrease its exposure to novel predators in its introduced populations, and reduce its post-autotomy susceptibility to predation during tail regeneration. This minor difference in the habitat preferences in the two *Lampropholis* species may contribute, in part, with a range of other behavioural traits (e.g. [29], [43], [44]), to the success of *L. delicata* as an invasive species.
